# Cost and impact of scaling up female genital mutilation prevention and care programs:  Estimated resource requirements and impact on incidence and prevalence

**DOI:** 10.1371/journal.pone.0244946

**Published:** 2021-01-28

**Authors:** Itamar Katz, Rachel Sanders, Maria Nadia Carvalho, Howard S. Friedman, Berhanu Legesse, William Winfrey, Nafissatou Diop

**Affiliations:** 1 UNFPA, New York, NY, United States of America; 2 Avenir Health, Takoma Park, MD, United States of America; Murdoch University, AUSTRALIA

## Abstract

**Purpose:**

SDG 5.3 targets include eliminating harmful practices such as Female Genital Mutilation (FGM). Limited information is available about levels of investment needed and realistic estimates of potential incidence change. In this work, we estimate the cost and impact of FGM programs in 31 high burden countries.

**Methods:**

This analysis combines program data, secondary data analysis, and population-level costing methods to estimate cost and impact of high and moderate scaleup of FGM programs between 2020 and 2030. Cost per person or community reached was multiplied by populations to estimate costs, and regression analysis was used to estimate new incidence rates, which were applied to populations to estimate cases averted.

**Results:**

Reaching the high-coverage targets for 31 countries by 2030 would require an investment of US$ 3.3 billion. This scenario would avert more than 24 million cases of FGM, at an average cost of US$ 134 per case averted. A moderate-coverage scenario would cost US$ 1.6 billion and avert more than 12 million cases of FGM. However, average cost per case averted hides substantial variation based on country dynamics. The most cost-effective investment would be in countries with limited historic change in FGM incidence, with the average cost per case averted between US$ 3 and US$ 90. The next most effective would be those with high approval for FGM, but a preexisting trend downward, where cost per case averted is estimated at around US$ 240.

**Interpretation:**

This analysis shows that although data on FGM is limited, we can draw useful findings from population-level surveys and program data to guide resource mobilization and program planning.

## Introduction

SDG Goal 5.3 aims to eliminate harmful practices, including FGM [1]. Analysis indicates that as many as 200 million women currently alive have undergone FGM, and 68 million girls are at risk by 2030 if current rates persist [2,3]. This practice has no known health benefits, but can result in a range of physical and mental health consequences, including but not limited to pain, bleeding, infection, complications in childbirth, issues with sexual function, and psychological consequences, and even death [4,5].

Community empowerment programs to change social norms provides opportunities to change these practices [6–8]. These programs address social norms, reflecting that what communities believe and how they expect community members to behave are central to reducing FGM. They also include a range of actors, from religious and political leaders to family members, and are crafted to reflect the context in which they are implemented. Limited data is available about the direct impact of prevention programs; however, a number of studies have shown impact on attitudes and beliefs [9,10].

To date, no estimates have been published showing the levels of investment needed to achieve substantial reductions in FGM. To provide more data on the investment needs for FGM programs, we estimated the cost of scaling up prevention, protection, and care and treatment programs in 31 low- and middle-income countries with high rates of FGM (S1 Appendix). The team also estimated the potential impact of those investments, based on the theory of social change that suggests that as social norms and the perception of community standards change, the incidence of FGM will be reduced [11]. Scenarios were developed to represent high and moderate levels of scaleup, and sensitivity analysis was performed on key variables.

## Methods

### Interventions and unit costs

Interventions were defined based on social norms work being done by the UNFPA-UNICEF Joint Programme on Elimination of Female Genital Mutilation [12] in Ethiopia, Guinea, Burkina Faso, and Djibouti, Tostan’s Community Empowerment Program [13], and other FGM programs identified through systematic reviews [14–17].

Building on these findings, the interventions costed in the prevention area were community-based empowerment and prevention programs and mass media, along with capacity building and material development costs. These programs involved intensive interpersonal communication, the use of a range of mechanisms to reach its target population, and training of community leaders. Practitioners help people identify harmful norms and facilitate reflections about the extent to which those norms and practices affect people’s health, happiness, and wellbeing, and support the community to identify and implement strategies to change those norms [18–20].

For each community receiving direct programs, it was assumed that an additional three communities will be sensitized indirectly, via inter community conversations and meetings with directly contacted community members, including public declaration gatherings, interactions with prevention trained health workers, and possible exposure to mass media which would not be restricted to communities receiving direct programming [21]. Additional details can be found S2 Appendix.

Protection programs included development of legislation and policies in countries where no legal framework exists (eight countries noted in S1 Appendix), along with mobile courts which combine awareness raising and law enforcement, and capacity building for legal personnel. Care and treatment interventions are assumed to target women with FGM type 3 (infibulation) [3], and consist of psychosocial support, as well as the training for health workers on management of FGM [22].

In most cases, unit costs were only available for a limited number of countries. These were converted to estimates for other countries to take into account the differences in purchase prices and salaries by applying a ratio based on the GDP (PPP) in the country being estimated compared to the country where the unit cost originated [23].

### Estimating national FGM intervention costs

For each intervention or activity, national costs were estimated by multiplying the target population by the population in need (PIN) of services, the coverage in each year, and the cost per person reached. Target populations are defined as the population that could receive the services, while the PIN is the proportion of that group who should receive it. Please see Table 1 for details on targeted populations for each activity.

For each intervention:

TargetpopulationXPINXCoverage=Numberreached

NumbersreachedXUnitcost=Totalactivitycost

**Table 1:** Interventions and assumptions.

	Intervention	Target Population	Population in need
**Prevention**	Community empowerment prevention programs	Communities, calculated as total population/average community size of 600 persons	% of communities where >50% of the population has positive views of FGM, estimated at more than 722,000 communities in 2020
	Mass and social media		
	Health provider training on prevention	Health providers	% of providers working in communities where >50% of the population has positive views of FGM
**Protection**	Legislation and policy development	Countries with no legislation prohibiting FGM	100% for those countries without legislation, zero for others
	Mobile courts	Communities, calculated as total population/average community size of 600 persons	% of communities where >50% of the population has positive views of FGM
	Capacity building for legal personnel	Legal personnel, one event per country annually	100%
**Treatment and care**	Psychosocial support	Women having a first birth	% who have experienced FGM type 3
	Capacity building for health providers on treatment and care	Health providers	% of providers working in communities where >50% of the population has positive views of FGM

Results are presented in 2020 US dollars, with no inflation or discounting applied.

### Program support costs

Program costs are the costs of the support work needed to ensure a high-quality FGM prevention, protection, and care and treatment program, including the program management, supervision, monitoring and evaluation, transport, communications, and safety in conflict areas for some countries. We applied percentages over and above the costs of program implementation, as seen in Table 2.

**Table 2:** Program support costs.

Cost type	Value over and above service delivery costs	Source/notes
Program-specific human resources	1.0%	R4D Above Service Delivery Costs review [24]
Supervision	2.0%	
Transport	2.0%	
Communications and media	1.0%	
Monitoring and evaluation	7.5%	Average of range specified by donors of 5–10%
General program management	12.0%	UNFPA implementers overhead
Safety in conflict areas	1.0%	Program budget data; applied in countries where US State Department provides hazard pay
**Total**	**26.5% - 27.5%**	Variable based on safety levels

### Scenarios analyzed

Two scaleup scenarios were analyzed: a high-coverage scaleup scenario in which 100% of communities with majority (>50%) approval of FGM would be reached with either direct or indirect community prevention programs by 2030, and a moderate scenario in which 50% of communities with majority approval would be reached with either direct or indirect prevention programs. This will reach communities with more than 90% of the FGM burden in countries with relatively high approval rates overall, while countries with lower approval rates will also need to use geographic or other targeting to ensure they are reaching the highest priority communities. Similar coverage targets were applied to the care and treatment and mobile court programs for each scenario, while legislation programs were the same in each scenario since they were fixed costs for legislation development and dissemination. These scenarios were applied to all countries in this analysis; for countries wishing to customize their own scenarios, the tools to do so have been made available at https://impact40.org/.

### Estimates of prevalence and incidence of FGM

We calculated the incidence of FGM for children aged 0–14 using a multistage process seen in S3 Appendix. The age-specific prevalences of FGM are the sum of incidences at each age and year previous to current year. Note that the age-specific incidences are appropriately lagged by year(s) to assure that the correct incidence is applied [25].

“a” is the age of the girl for which we are calculating the prevalence.

“t” is the year for which we are calculating the prevalence.

$$Prevalence_{a,t}=\sum_{i=0}^{a}Incidence_{i,t-(a-i)}$$

The prevalences for women aged 15–49 are assumed to not change as a cohort ages.

Mathematically:

$$Prevalence_{a}{}_{,t}=Prevalence_{a}{}_{‐1,t‐1}\left(\mathrm{for}\;a>=15\right)$$

The adult prevalence will change over time as the children (where prevalence is changing) age into the adult age groups.

### Estimating impact of community programs

The impact of community programs was calculated as follows.

1. A regression was run to calculate logistic equation coefficients that were used to calculate probabilities that a daughter is cut. The regression-dependent variables were women’s support/nonsupport of FGM, average community support, and control factors including age, wealth, education, urban/rural location, and religion.
2. Women’s support status was changed based on the effectiveness of programs on changing attitudes. We assumed that the impact of FGM programming is largely the result of changes in the attitudes of individuals leading to changes in community norms [26]. A negative attitude of a mother toward the practice of FGM will lead to a lower probability that her daughter will be cut. In addition, a negative average community attitude toward the practice will lead to a lower probability that a daughter will be cut, independent of the mother’s attitude [17]. The degree to which these factors will influence the probability that a daughter will be cut is country specific.
  The effect of programs was quantified as effect sizes for direct beneficiaries and the indirectly sensitized communities. The direct effect of programs was modeled in the estimates as being 71.0% [27] effective in changing the attitudes of FGM supporters. The indirect effect of programs was assumed to be via sensitization in neighboring communities of those that are direct beneficiaries of the interventions, and was modeled using an effect size of 44.6% [28].
3. The new probability of a daughter being cut was calculated by using the regression coefficients applied to a specific country dataset, with the women’s attitudinal changes adjusted via the effect sizes above to reflect the effect of the women’s attitude changes on community support. The country-specific regression coefficients were used to model estimates of the probability that a daughter would be cut under different estimates of individual- and community-level support. Note that community levels of support changed as a result of the changes to individual-level support (i.e., we recalculate the community levels after the individual levels are changed). This multi step process was needed as data is not available about the direct effect on FGM of prevention programs.
4. The impact of the program on cutting practices was calculated as the difference between the original probability of being cut minus the new probability divided by the original probability of being cut.

We assumed that interventions are applied only in communities where there is more than 50% continued support for the practice of FGM [29]. For each of the white cells in Table 3, we calculated a probability that a woman in a community has a daughter who is cut. Using this, we are able to calculate overall national-level probabilities that a woman has a daughter who is cut based on the coverage of the intervention (i.e., % of communities with greater than 50% support who receive the intervention) and the number of communities who receive the indirect impact, which we translate into a coverage based on the coverage of the direct impact (i.e., number of communities multiplied by coverage of communities receiving direct impact) as in the example in S4 Appendix.

**Table 3:** Distribution of intervention groups.

	Community receives direct impact of intervention	Community receives indirect impact of intervention	Community receives no intervention
Community has greater than 50% support for continuing the practice	Interventions modeled	Interventions modeled	Interventions modeled
Community has less than 50% support for continuing the practice	No interventions modeled	No interventions modeled	Diffusion effects modeled

The assumption is that once opinions have been changed to be negative on FGM, this opinion change remains over time and does not revert to approval. We also assume that the intervention continues to change community norms after implementation. For that reason, we examine the impacts on FGM cases through an additional generation of girls in order to recognize the longer-term impacts of the intervention, although costs are only accrued at the time of intervention (during the period 2020–2030).

## Results and discussion

### Total costs

The high-coverage scenario would imply reaching nearly 150,000 communities with direct prevention and community empowerment programs, and assumes that an additional 450,000 communities would be reached indirectly, as well as providing psychosocial support to more than 1.9 million women who had FGM. The total cost for 2020 through 2030 would be about US$ 1 billion for the Middle East and North and Sub-Saharan Africa, with Asia adding another US$ 2.3 billion. The moderate-coverage scenario would cost around US$ 1.6 billion for the years 2020–2030.

Asia is the largest cost contributor at 68% of the costs, followed by the Middle East and North Africa region (24% of the total), and Sub-Saharan Africa (8%).

Materials development and legislative action costs were front-loaded. In addition, the pool of communities with majority positive views of FGM shrinks over time based on historic trends. As a result, the resource requirements are slightly lower in later years.

The majority of costs were associated with prevention programs, at 63% of costs, followed by program support costs (20%). Care and treatment and prevention programs account for approximately 11% and 5%, respectively.

### Cases averted

We estimate the number of cases of FGM averted by comparing the number of cases of FGM in the high- and moderate-coverage scenarios where both interventions and historic trends affect incidence to a counterfactual where only historic rates of change affect incidence. Substantial numbers of cases of FGM could be prevented by these programs, with the high-coverage scenario estimated to avert 4.6 million cases of FGM in the years 2020–2030 while the intervention is underway, and an additional 19.8 million among the subsequent cohort of girls during the years 2031–2050. This results in a total of 24.4 million cases averted in the high-coverage scenario while the moderate-coverage scenario would avert 12.1 million cases. In both cases, the average cost per case averted is around $134. The intervention-based reductions are in addition to the reductions in incidence associated with historic trends due to previous interventions, as well as education and other social and economic dynamics, which are estimated to avert an additional 46 million cases of FGM by 2050 (historic trends and the interventions discussed here could avert FGM for 45% of girls at risk by 2050) [30,31].

These gains increase over time, implying that examining a longer time horizon would show even greater results. The average cost per case averted hides a wide range of variation based on country setting and assumes constant costs over time; see the discussion below for more details.

### Sensitivity analysis

This analysis was limited by data availability in a number of areas. As seen in Table 4, we performed sensitivity analysis to understand more about how issues with our assumptions could affect results, focusing on assumptions around indirect sensitization of communities and duration of effect of the prevention programs, as well as the herd effects of the interventions that could reach communities that are neither directly nor indirectly sensitized through the intervention and costs in high-security settings and the implications of including countries with limited data available.

**Table 4:** Sensitivity analysis results.

Parameter	Primary assumption	Alternative assumptions	Impact of sensitivity analysis on cost results	Impact of sensitivity analysis on cases estimates
Communities sensitized indirectly	3 communities sensitized indirectly	2 communities sensitized indirectly	No change	10% fewer cases of FGM prevented
Spillover effects	No impact in communities where views of FGM are already relatively negative	Spillover effects modeled in all communities	No change	Cases of FGM reduced by 18–25% more, depending on effectiveness assumed for communities not reached directly
Post-intervention effects	Effects of prevention programs continue at similar levels beyond the final year of implementation	50% effective No post-intervention effect	No change	Reduction to 22.6 million cases of FGM averted Reduction to 18.6 million cases of FGM averted
Costs of working in insecure zones	Additional 1% of program implementation costs	Additional 50% of program implementation costs	5% increase in total costs	No change
No historic trend in Indonesia	3% annual reduction in community approval	Zero historic trend in Indonesia due to limited data	US$ 400 million increase in total costs	No change
All listed above	As described in body of document	Optimistic/best case	No increase in costs	More than 30 million cases averted
		Pessimistic/worst case	Increase to around US$ 4 billion	Reduction to around 17 million cases averted
Data availability	Only examined cost and impact of countries with available DHS or MICS	Removed 9 data poor countries from analysis	Cost of program would decrease to US$ 940 million	Impact would fall to 18.4 million cases averted

The COVID-19 pandemic could also affect these estimates by delaying the scaleup of prevention efforts due to lock-down terms that preclude social mobilization events, and diverting the attention and efforts of health and social programs instead to COVID-19 control. Assuming a later start of programs (i.e., using a 2-year delay in 2020 and 2021) in many countries as a result of these factors, and resulting lower program coverage achievements by 2030, it is estimated that cases averted between 2020–2030 would be reduced by 33%. At the same time, if all communities were targeted, rather than just majority approval communities, there would be a faster decline in FGM incidence and prevalence, but it would imply higher costs and lower cost-effectiveness

Although the sensitivity analysis highlights the importance of various assumptions, the overall conclusions on the importance of changing social norms around FGM, and the potential of prevention programs to achieve these goals remain consistent.

## Discussion

It is important to understand the cost drivers that lead to variation in costs between countries. Overall population size is a large factor–all else being equal, the larger the population, the larger the costs. For those countries with higher GDP, the costs of reaching a person or community with services are higher. Prevention program costs are higher in countries where more communities have majority positive views of FGM, primarily due to the costs of reaching higher numbers of communities with prevention programs. Countries that do not yet have legislation regulating FGM will have higher protection costs. With higher prevalence, and with higher proportions of FGM type 3, come higher care and treatment costs. The five highest-cost countries and their primary cost drivers are shown in Table 5.

**Table 5:** National cost drivers.

	Population size	GDP	Proportion of communities with positive views of FGM	High proportion of FGM type 3
Indonesia	✔	✔	✔	
Egypt	✔	✔	✔	
Sudan	✔		✔	✔
Nigeria	✔	✔		
Mali			✔	

Looking at countries based on their level of support for FGM and the historic trends around FGM (i.e., whether existing prevention programs and socioeconomic change have already jump-started a reduction in FGM incidence) gives us four groups to consider: strong historic trend and high approval, limited historic trend and high approval, strong historic trend and low approval, limited historic trend and low approval.

If we consider the cost and impact results for these country groups, we can prioritize in cases of scarce resources. Unsurprisingly, our findings indicate that it is most cost effective and impactful to invest in the countries that have limited historic change and higher approval ratings (including Djibouti, Eritrea, Gambia, Guinea, Mali, Mauritania, Nigeria, and Yemen), with the average cost per case averted being between $3 and $90. In 2020, countries with limited historic reduction trends represent around 48% of the burden. Where countries still have many communities with majority approval, but a pre-existing historic trend downward, it is still cost-effective, but impacts attributable to new prevention programs are lower, leading to slightly less cost-effective results (in the range of $240 per case averted). The average cost per case averted is around $2,000 (mostly fixed costs of running programs and care costs incurred due to past levels of FGM) in countries where approval is already low and a strong historic trend exists.

### Comparisons to existing spending

Although there are no current estimates that aggregate global spending on FGM reduction efforts, the UNFPA-UNICEF Joint Programme on FGM estimated it would spend approximately US$ 19 million per year during its Phase 3 (2018–2021) [32], indicating a substantial resource gap of nearly US$ 280 million per year to implement the prevention, care and treatment, and protection programs if these levels continue and there are limited other investments in this area.

### Limitations

This analysis highlights the lack of research on FGM program effectiveness and cost. As such, we relied on one study for the estimates of direct benefits and an internal program implementer results analysis for the estimates of indirect benefits. Unit costs are based on limited country datasets representing programs in five countries (Senegal, Ethiopia, Guinea, Burkina Faso and Djibouti) and program costs are based on generalized above service delivery costs for other sexual and reproductive health programs in lower and middle income countries, but insufficient information was available to allow differentiation by individual country or region. Other data gaps include the lack of sufficient survey data in nine countries to allow for incidence estimates based on recent trends. Similarly, assumptions around sensitization in neighboring communities have a limited evidence base.

Due to the nature of the analysis, which covered 31 diverse countries, standardized assumptions such as community size, facilitator compensation, and program structure had to be made to allow estimates of the costs of activities. However, in practice, there will likely be more variation in the implementation of programs that would cause costs to differ. There may also be changes to the cost structure over time, and as programs scale up, including both decreases due to economies of scale, and increasing marginal costs as countries work with harder to reach communities. Given the limitations of predicting these changes, this analysis has assumed constant unit costs.

Additionally, the impact analysis was an indirect process, looking first at the impact of prevention programs on community and individual views, and then at the impact of changing views on actual practice. This should be a focus of future research; as more data becomes available about the direct effect of prevention programs on FGM rates, it will be possible to analyze the effect of scaling up prevention programs directly.

Although evidence exists that education, urbanization, mobility, and other social trends affect decisions around FGM, this analysis relies on past historic trends without attempting to predict the role that future educational attainment and other social dynamics will play in reducing FGM.

We were unable to estimate changes to standard health outcomes like disability-adjusted life years as there are no standardized disability weights associated with FGM. This limits the potential for comparing FGM programs to other health programs. We hope this work will lead to more rigorous program evaluations and incorporation of FGM into Global Burden of Disease estimates.

Given the limitations, these estimates should be interpreted as a discussion point and advocacy tool to emphasize the investment needs for FGM programming, but would require more country-level input to be used to set targets for individual countries.

## Supporting information

S1 Appendix Countries include in analysis. (DOCX)

S2 Appendix Unit cost assumptions. (DOCX)

S3 Appendix Estimating incidence. (DOCX)

S4 Appendix Impact calculations. (DOCX)
