# Supplementary material for: Cost and impact of scaling up female genital mutilation prevention and care programs: Estimated resource requirements and impact on incidence and prevalence
Source: PLoS One. 2021 Jan 28;16(1):e0244946. doi: 10.1371/journal.pone.0244946 (PMC7842986; doi:10.1371/journal.pone.0244946)
Supplement: S1 Appendix — (DOCX) [file pone.0244946.s001.docx]

## S1 Appendix. Countries include in analysis.

| **Country** | **Survey** |
| --- | --- |
| Benin | 2014 MICS |
| Burkina Faso | 2010 DHS |
| Cameroon* | 2011 DHS |
| Central African Republic | 2010 MICS |
| Chad | 2014 DHS |
| Côte D’Ivoire | 2011 DHS |
| Djibouti* | 2006 MICS |
| Egypt | 2015 DHS |
| Eritrea* | 2002 DHS |
| Ethiopia | 2016 DHS |
| Gambia | 2010 MICS |
| Ghana | 2011 MICS |
| Guinea | 2018 DHS |
| Guinea-Bissau | 2014 MICS |
| Indonesia*^+^ | 2016 RISKEDAS |
| Iraq*^+^ | 2011 MICS |
| Kenya | 2014 DHS |
| Liberia*^+^ | 2013 DHS |
| Maldives^+^ | 2016 DHS |
| Mali^+^ | 2018 DHS |
| Mauritania | 2015 MICS |
| Niger* | 2012 DHS |
| Nigeria^+^ | 2018 DHS |
| Senegal | 2016 DHS |
| Sierra Leone^+^ | 2017 MICS |
| Somalia | 2011 MICS |
| Sudan^+^ | 2014 MICS |
| Togo | 2013 DHS |
| Uganda* | 2016 DHS |
| Tanzania | 2015 DHS |
| Yemen* | 2013 DHS |

* These countries had insufficient data for country-level incidence calculations. Incidence change estimates were based on trends for proxy countries.

^+^ These countries do not have legislation against FGM at the time of this publication.
