# Supplementary material for: Cost and impact of scaling up female genital mutilation prevention and care programs: Estimated resource requirements and impact on incidence and prevalence
Source: PLoS One. 2021 Jan 28;16(1):e0244946. doi: 10.1371/journal.pone.0244946 (PMC7842986; doi:10.1371/journal.pone.0244946)
Supplement: S2 Appendix — (DOCX) [file pone.0244946.s002.docx]

## S2 Appendix. Unit cost assumptions.

| **Intervention** | **Details** | **Source** |
| --- | --- | --- |
| **Prevention** | | |
| **Development of training material** | Training materials for health workers, mobile courts, district officials, religious leaders, community management, facilitator training, and refresher training, | Consultations with FGM program implementers at UNFPA |
| **Community programs** | |  |
| **Facilitator training** | DSA for 1 facilitator per community for 10-day training; consulting fee (country-specific) and DSA for 2 trainers for 10 days training and 2 days preparation | UNFPA community partners in Ethiopia, Guinea FGM survey November 2017, Tostan, Senegal |
| **Refresher training** | DSA for 2-day training per community facilitator; DSA and consultant fee (country-specific) for 2 days training and 1 day preparation for 2 trainers (per 25 trainees) | UNFPA FGM team; UNFPA community partner Burkina Faso and Ethiopia |
| **Inter-community meetings** | DSA for 1 meeting per month for 6 months with 2 participants per community | Consultations with FGM program implementers at UNFPA |
| **Training: community management committees** | DSA for 5-day training for 5 participants per community; DSA and consulting fee for 2 trainers (per 25 participants) for 5 days of training and 2 days preparation |  |
| **Training: religious leaders** | DSA for 5-day training for 2 religious leaders per community; DSA and consulting fee for 2 trainers (per 25 trainees) for 5 days training and 2 days preparation |  |
| **Training: district officials** | DSA for 5 days training for 30 participants; DSA and consulting fee for 2 trainers (per 25 trainees) for 5 days training and 2 days preparation |  |
| **Public declaration of FGM abandonment** | Cost per community based on country-specific costs | Tostan, Senegal; UNFPA community partner Ethiopia, Djibouti |
| **Radio** | Resource Needs Model; cost per person for HIV prevention radio programming | UNAIDS Resource Needs Model |
| **Training for health workers** | DSA for 50 trainees for 5-day training; trainer fee and DSA for 4 trainers and support staff for 28 person days (5 days training and 2 days) preparation | UNFPA FGM team; UNFPA community partner Burkina Faso |
| **Community programs** | |  |
| **Community outreach** | |  |
| **Material** | Cost per person |  |
| **Community facilitator** | Monthly rate for 1 full-time facilitator per community | Tostan, Senegal |
| **Community supervisor** | For 1 supervisor per 10 communities: 10% FTE of a supervisor | UNFPA community partner Ethiopia |
| **Community supervisor car & transport costs** | For 1 supervisor per 10 communities: Vehicle: 10% of vehicle cost; Fuel cost 1,467 L per year | UNFPA community partner Ethiopia |
| **Protection: legislation** | |  |
| **Drafting a bill & producing evidence-based research on social norm and FGM** | 1 consultant per country (cost not country-specific) | Consultations with FGM program implementers at UNFPA |
| **Two 1-day consultation workshops** | 100 participants (based on cost per participant per day) | Country-level workshops in Botswana, Georgia, Indonesia, Kenya, Lebanon, and Zambia and the H6 joint program (a joint partnership of UNFPA, UNAIDS, UNICEF, WHO, UN Women, World Bank) |
| **Advocacy among journalists for PR purposes** | 3 per country per year (cost not country-specific) | Consultations with FGM program implementers at UNFPA |
| **Protection: enforcement** | |  |
| **Capacity development workshops** | 2 3-day workshops with DSA for 50 participants | Consultations with FGM program implementers at UNFPA |
| **Mobile courts** | 12 visits, DSA for 1 lawyer and 1 judge |  |
| **Treatment and care** | |  |
| **Psychosocial support** | Country-specific per-person cost based on individual support, group-based support, home visits, etc. Assumed to be provided to women with Type 3 FGM having a first birth. | FGM survey November 2017: Mauritania, Côte d'Ivoire; HIV/AIDS Program Sustainability Analysis Tool (HAPSAT), Guyana and Sierra Leone [1] |
| **Capacity building for health providers** | DSA for 50 trainees for 5-day training; trainer fee and DSA for 4 trainers and support staff for 28 person days (5 days training and 2 days) preparation | UNFPA FGM team; UNFPA community partner Burkina Faso |

References

1. Katz I, Altman D, Osika J, et al. Sustainability analysis of HIV/AIDS services: HAPSAT Guyana and Sierra Leone. Bethesda, MD: Health Systems 20/20 project, Abt Associates Inc. and National AIDS Program; 2011.
