# Supplementary material for: Cost and impact of scaling up female genital mutilation prevention and care programs: Estimated resource requirements and impact on incidence and prevalence
Source: PLoS One. 2021 Jan 28;16(1):e0244946. doi: 10.1371/journal.pone.0244946 (PMC7842986; doi:10.1371/journal.pone.0244946)
Supplement: S3 Appendix — (DOCX) [file pone.0244946.s003.docx]

## S3 Appendix. Estimating incidence.

1. For children aged 0-14 we tabulated the age-specific incidence of FGM from Demographic and Health Survey (DHS) or Multiple Indicator Cluster Survey (MICS) datasets for the year of the survey based on responses of a mother to queries about whether her child has been cut, and if yes, at what age she was cut.
2. We calculated a time trend for FGM reduction based on a tabulation of the historical age-specific incidences for the age at which incidence of FGM is greatest in a country. This age varies by country – in most West African countries it is children less than 1 year old, while in East and North Africa the ages range mostly from 5 to 12 years old.
3. We calculated an intervention-specific reduction based on the regression described in the subsequent section. This reduction is spread across 12 years (for consistency with cost estimates).
4. We applied a year-to-year incidence reduction at every age, calculated as the sum of the historical trend (step 2) and the intervention-based reduction (step 3). Note that the historical trend is applied at every year between the year of the latest survey and the end of the projection period. The intervention-based reduction is applied only to the years 2020 through 2030.
