# Supplementary material for: Cost and impact of scaling up female genital mutilation prevention and care programs: Estimated resource requirements and impact on incidence and prevalence
Source: PLoS One. 2021 Jan 28;16(1):e0244946. doi: 10.1371/journal.pone.0244946 (PMC7842986; doi:10.1371/journal.pone.0244946)
Supplement: S4 Appendix — (DOCX) [file pone.0244946.s004.docx]

## S4 Appendix. Impact calculations.

Since girls are likely to be cut at different ages in different countries, we used DHS or MICS surveys to identify the most probable age to be cut, designated below as “a”, in each country. We then defined a woman as having a daughter who was cut as a woman who had a daughter or daughters between age “a” and five years older “a+5”. In the regression we included only women who had daughters between the age of “a” and “a+5”.

Communities were defined as sampling clusters in the surveys. The sampling clusters are not perfect measures of a community as they do not necessarily correspond to an administrative unit, nor are they are necessarily uniform in terms of ethnic or other important cultural factors. On the other hand, the clusters are a group of households that are near to one another (geographically compact). The level of support for the practice of FGM was defined as the percent of all interviewed women aged 15-49 (not just those with daughters) in the cluster who believed that the practice of FGM should be continued.

The following charts work through a notional example. Every country/survey will have different distributions and probabilities associated with it.

**Table 1. Percent distribution of intervention groups**

|  | Community receives direct impact of intervention | Community receives indirect impact of intervention | Community receives no intervention |
| --- | --- | --- | --- |
| Community has greater than 50% support for continuing the practice | 2.1% | 6.2% | 0.0% |
| Community has less than 50% support for continuing the practice |  |  | 91.7% |

**Table 2. Probabilities of a daughter being cut based on DHS analysis**

|  | Community receives direct impact of intervention | Community receives indirect impact of intervention | Community receives no intervention |
| --- | --- | --- | --- |
| Community has greater than 50% support for continuing the practice | 0.087 | 0.113 | 0.228 |
| Community has less than 50% support for continuing the practice |  |  | 0.127 |

The post-distribution probability will be:

0.127*91.7% + 0.087*2.1% + 0.113*6.2% + 0.228*0.0% = 0.127

The percent reduction in probability of being cut (or alternatively, the percent reduction in incidence) is the difference between the baseline incidence and the endline incidence divided by the baseline value. In the case above, it would be the following:

%Reduction_Incidence = [ Prob(pre-intervention) – Prob(post-intervention) ] / Prob(pre-intervention)

Prob(pre-intervention) = 0.138

Prob(post-intervention) = 0.127

%Reduction_Incidence = (0.138 – 0.127) / 0.138 = 8%
